# Mediating Effect of Stress Recognition on the Effect of Generalized Anxiety Disorder on Smartphone Dependence

**DOI:** 10.3390/jcm12237359

**Published:** 2023-11-28

**Authors:** Se Ryeon Lee, Eun-Yeob Kim, Seunghan Ha, Jaeyoung Kim

**Affiliations:** 1 Division of Oncology/Hematology, Department of Internal Medicine, Korea University Ansan Hospital, Ansan-si 15355, Gyeonggi-do, Republic of Korea; logost@korea.ac.kr; 2 Research Institute for Skin Image, Korea University College of Medicine, Seoul 08308, Republic of Korea; key0227@korea.ac.kr; 3 Department of Medical Rehabilitation, School of Health, Chungbuk Health and Science University, Cheongju-si 28150, Chungbuk, Republic of Korea; 4 Department of Biomedical Sciences, College of Medicine, Korea University, Seoul 02841, Republic of Korea; 5 Core Research & Development Center, Korea University Ansan Hospital, Ansan-si 15355, Gyeonggi-do, Republic of Korea

**Keywords:** smartphone dependence, generalized anxiety disorder (GAD), stress recognition, adolescents, process-macro, mental health

## Abstract

The widespread adoption of the smartphone has led to both positive and negative consequences for adolescents’ mental health. This study examines the interplay between smartphone dependence (SPD), generalized anxiety disorder (GAD), and various mental health outcomes among Korean adolescents. Data from the 16th Adolescence Health Behavior Survey (2020), including 54,948 middle and high school students, were analyzed. Adolescents were categorized into three groups based on SPD severity. The GAD-7 scale assessed anxiety, and other factors such as subjective health recognition, happiness, weight control efforts, and body mass index (BMI) were considered. Adolescents with higher SPD exhibited lower academic performance, decreased happiness, and increased perception of stress. GAD levels were positively correlated with SPD, with higher SPD linked to more severe GAD symptoms. Additionally, higher SPD was associated with increased loneliness, sadness, and suicidal thoughts, plans, and attempts as well as a greater likelihood of habitual drug use. Gender differences revealed that females were more prone to sadness, hopelessness, and suicidal thoughts, while males exhibited higher rates of drug use. This study highlights the complex relationship between SPD, GAD, and mental health outcomes among Korean adolescents. Stress recognition was found to mediate the association between GAD and SPD. The process-macro result of the total effect between SPD on GAD and the direct effect of the SPD pathway on GAD was significant; thus, the stress recognition was mediated. Effective interventions should target stress management, especially among adolescents with high smartphone dependence, to mitigate the risk of mental health issues. These findings underscore the importance of addressing smartphone dependence and its impact on the mental well-being of adolescents.

## 1. Introduction

The widespread adoption of smartphones as indispensable tools in daily life has been exponential. In 2020, ownership rates reached 93.1% among Koreans overall and surged to 99% among Korean adolescents [1]. This technological advancement has facilitated various applications, transforming smartphones into vital devices for everyday activities, entertainment, and leisure. However, the accelerated integration of smartphones has brought about a concerning escalation in both psychological and physical side effects [2,3].

The National Information Society Agency’s report in 2014 highlighted a spectrum of issues arising from excessive smartphone usage, notably encompassing dependence, tolerance, withdrawal, impatience, and anxiety linked to negative emotions [4,5]. Among adolescents, the challenges in managing stress due to constraints in time and space have led to a reliance on smartphones, potentially leading to daily life difficulties [4,5,6,7,8].

Statistics reveal that a significant proportion of middle and high school students, approximately 34.3% and 28.7%, respectively, fall into the high-risk group for smartphone dependence (SPD), with a substantial 81.6% predominantly engaged with social network services (SNS) [9]. Notably, improper stress management among adolescents has been correlated with increased SPD and manifestations of addiction symptoms due to excessive internet usage [10].

The nexus between stress, generalized anxiety disorder (GAD), and smartphone over-dependence has been well established. Studies have suggested that internet addiction serves as a coping mechanism to alleviate stress, especially among elementary school students [11]. Given its prevalence across various age groups, including adults [12,13,14], there is a critical need to delve deeper into understanding the primary causes of GAD and stress concerning SPD.

Adolescence, a critical phase of rapid physical and mental growth, lacks a fully established adult self-identity. Excessive smartphone usage during this phase elevates the risk of adverse outcomes, including addiction, anxiety, isolation, and impulsive behavior [12,13]. These negative impacts extend to behavioral problems such as drug involvement, violence, and sexual issues [13], contributing to the notably low happiness index among Korean children compared to other countries [14]. Many adolescents grapple with daily stress, making it a pressing concern [15].

Generalized anxiety disorder (GAD), characterized by persistent worry and anxiety, particularly affects adolescents in their mid-teens to early twenties, intensifying during periods of high stress and complicated by various factors [16,17,18,19,20,21]. The inability to control anxiety may lead to chronic conditions, resulting in decreased concentration, anxiety disorders, and disrupted sleep patterns, significantly impairing adaptive function [22,23,24,25].

This study aimed to examine the mediating effect of stress perception on the relationship between mental anxiety in adolescents and smartphone addiction. Additionally, the results of this study are intended to contribute to the research foundation for promoting adolescent health. Different factors that affect smartphone use among adolescents were identified according to their demographic or health characteristics. We analyzed how SPD affects health behavior and whether there should be a correlation between depression and suicidal thoughts due to SPD.

## 2. Material and Method

### 2.1. Study Designs and Sampling

This study used the data of the 16th (the year 2020) “Adolescence Health Behavior Survey”, which is joint research by the Ministry of Education, the Ministry of Health and Welfare, and the Korea Disease Control and Prevention Agency (IRB Approval No. 117058). The sampling process involved stratification of the population, distribution of samples, and extraction of samples. All students from the selected classes, which were determined through the stratification process, were the target population for sampling and recruitment. On the survey day, long-term absentees, special-needs children, and students with communication disorders were excluded from participation. The survey period spanned from 3 August 2020 to 13 November 2020. A total of 800 schools, comprising 400 middle schools and 400 high schools, were included in the study. We enrolled 54,948 adolescents who were divided according to the degree of SPD into a normal group (*n* = 41,173), a self-control failure (SCF) group (*n* = 12,142), and serious consequences (SC) group (*n* = 1633). Smartphone dependence among males is classified into three groups: a normal group (*n* = 22,521, 79.4%), an SCF group (*n* = 5239, 18.5%), and an SC group (*n* = 593, 2.1%). Among females, the classification included a normal group (*n* = 18,652, 70.1%), an SCF group (*n* = 6903, 26.0%), and an SCF group (*n* = 1040, 3.9%).

### 2.2. Measures

The dependent variable was the degree of smartphone dependence. Demographic characteristic variables were height (m), weight (kg), age, grade score, grade, parental education, and economic status. Subjective health recognition variables were subjective health, subjective body type, subjective happiness, weight control effort, and BMI. Mental health variables were subjective stress recognition, GAD, loneliness/sadness/hopelessness/suicidal ideation, and planning and attempting/non-therapeutic drug experience.

The SPD scale (https://www.iapc.or.kr/kor/PBYS/diaSurvey.do?idx=8 (accessed on 1 September 2023)) tool uses ten questions with a total score of 40 points ≤22 points—normal, 23–30 points—SCF, and ≥31 points—SC. The higher the scores, the higher the tendency of SPD. As a result of analyzing the internal agreement between the questions on this scale, Cronbach’s α was found to be 0.915. The GAD 7 scale developed by Spizer (2006) was used in the adolescent health behavior survey [25]. It consists of seven questions on a 4-point Likert scale (0 = not at all agree; 3 = strongly agree) related to the anxiety or worry experienced in the last two weeks. The higher the combined score, the higher the anxiety tendency. The response result of the GAD 7 scale, often used as a primary screening tool for GAD in medical institutions, was selected to be suitable for estimating the general anxiety level of sedentary adolescents. As a result of analyzing the internal agreement between the questions on this scale, Cronbach’s α was found to be 0.898. Obesity was defined as a body mass index (BMI) > 25 kg/m^2^. Obesity was measured using the criteria of the WHO Asia Pacific region and Korean Society of Obesity.

### 2.3. Data Analysis

We conducted descriptive statistics. Categorical variables were expressed as frequency (percentage), and continuous variables were expressed as mean ± SD. The chi-square test was used to analyze nominal variables, and the Kruskal–Wallis test was used to analyze continuous variables. For the hypothesized model in this study, the process-macro version 3.5 model 4 of Hayes (2013) was used to confirm the total direct and indirect effect of stress recognition mediating the effect of GAD on SPD [26]. SPSS/WIN version 25.0 program (IBM Corp., Armonk, NY, USA) was used for statistical analysis. Process-macro is a method proposed by Hayes (2013) to evaluate the significance of indirect effects through bootstrapping [26]. Mediation analysis is a method to test whether the independent variable indirectly influences the dependent variable through a mediator. In the process-macro analysis, the bootstrap sample was 5000, and the confidence interval was 95%.

## 3. Results

### 3.1. Smartphone Dependence and General Characteristics

The characteristics of the study participants by the degree of SPD are shown in Table 1. It shows that the height was shorter in the normal group (171.04 ± 7.68 cm) than in the SCF (171.32 ± 7.28 cm) and SC (171.94 ± 7.44 cm) group (*p* = 0.016). In males, the age of SPD was higher in the SC (15.54 ± 1.74 years) and SCF groups (15.26 ± 1.73 years) than in the normal group (15.07 ± 1.76 years) (*p* < 0.001). In females, 9.9% were obese in the normal group, 10.1% in the SCF group, and 8.8% in the SC group (*p* = 0.419). This corresponds to an overall obesity of approximately 23.1% in males and 9.6% in females. “High or middle” grade in academic performance was considered to correlate with low SPD. Academic performance was 38.7% in the normal group, 32.5% in the SCF group, and 30.0% in the SC group (*p* < 0.001). High school grade-level or higher SPD was found to be 46.9% in the normal group, 50.1% in the SCF group, and 55.3% in the SC group (*p* < 0.001). An economic status of “middle or high”, which gradually reduced SPD, was 87.6% in the normal group, 85.0% in the SCF group, and 78.8% in the SC group (*p* < 0.001). In females, the years of SPD were higher in the SC group at 15.26 ± 1.66 years and in the SCF group at 15.21 ± 1.69 years than in the normal group at 15.01 ± 1.77 years (*p* < 0.001). Middle or high school grades were low, according to SPD. Middle or high school grades were 38.4% in the normal group, 30.8% in the SCF group, and 26.5% in the SC group (*p* < 0.001). High school grade-level or higher SPD was 45.7% in the normal group, 49.9% in the SCF group, and 8.7% in the SC group (*p* < 0.001). A family income status of “middle or high” was 88.0% in the normal group, 84.5% in the SCF group, and 82.7% in the SC group (*p* < 0.001) (Table 1).

### 3.2. Smartphone Dependence and Weight Control, Recognition

Weight control and recognition results according to the degree of SPD show that males are healthier than females. Males had scores of 4.08 ± 0.88 in the normal group, 3.81 ± 0.91 in the SCF group, and 3.64 ± 1.12 in the SC group (*p* < 0.001). According to subjective happiness, the normal group (3.99 ± 0.94) was happier than the SCF group (3.67 ± 0.95) and the SC group (3.37 ± 1.17) (*p* < 0.001). As a result of weight control efforts in the last month, the normal group made 52.6% effort to control weight, the SCF group made 50.3%, and the SC group made 47.9% (*p* < 0.001). In females, subjective health recognition was healthier in the SCF group at 3.59 ± 0.88 and in the SC group at 3.45±1.02 than in the normal group at 3.84 ± 0.87 (*p* < 0.001). According to subjective body type recognition, the normal group had scores of 3.24 ± 0.88, the SCF had scores of 3.28 ± 0.94, and the SC group had scores of 3.28 ± 1.01, considered as “slim” (*p* < 0.001). Subjective happiness revealed that the normal group (3.80 ± 0.95) was happier than the SCF group (3.50 ± 0.93) and the SC group (3.28 ± 1.01) (*p* < 0.001). The result of weight control efforts in the last month revealed that the normal group made 58.7% effort to control weight, the SCF group made 56.1%, and the SC group made 54.2% (*p* < 0.001) (Table 2). 

### 3.3. Smartphone Dependence and Mental Health

Mental health outcomes, according to SPD, show that in men, the usual stress recognition was lesser in the normal group (2.95 ± 0.96) than in the SCF group (3.23 ± 0.85) and SC group (3.54 ± 1.04) (*p* < 0.001). GAD was lower in the normal group (9.67 ± 3.63) than in the SC group (11.65 ± 4.40) and SCF group (14.10 ± 6.23) (*p* < 0.001). According to loneliness experience in the previous year, the normal group (2.13 ± 1.03) experienced less loneliness than the SCF group (1.02 ± 4.40) and SC group (2.83 ± 1.26) (*p* < 0.001). Experiences of sadness and hopelessness in the previous year were 17.8%, 26.3%, and 40.6% in the normal, SCF, and SC groups, respectively (*p* < 0.001). Suicide attempts in the previous year were 1.2%, 1.4%, and 4.9% in the normal, SCF, and SC groups, respectively (*p* < 0.001). Habitual drug experience (bond, butane gas, etc.) was 0.7%, 0.9%, and 2.9% in the normal, SCF, and SC groups, respectively (*p* < 0.001). In females, stress recognition shows that the normal group (3.24 ± 0.90) had less stress than the SCF group (3.52 ± 0.86) and SC group (3.85 ± 0.93) (*p* < 0.001). GAD was lower in the normal group (10.98 ± 4.21) than in the SCF group (13.14 ± 4.74) and SC group (16.02 ± 6.04) (*p* < 0.001). Loneliness experiences in the previous year show that the normal group (2.47 ± 1.04) experienced less loneliness than the SCF group (2.90 ± 0.98) and SC group (3.18 ± 1.13) (*p* < 0.001). Experiences of sadness and hopelessness in the previous year were 26.3%, 39.5%, and 54.6% in the normal, SCF, and SC groups, respectively (*p* < 0.001). Suicide thoughts during the previous year were 11.5%, 18.4%, and 29.8% in the normal, SCF, and SC groups, respectively (*p* < 0.001). Suicide plans in the previous year were 3.7%, 5.5%, and 10.2% in the normal, SCF, and SC groups, respectively (*p* < 0.001). Suicide attempts in the previous year were 2.4%, 3.4%, and 6.3% in the normal, SCF, and SC groups, respectively (*p* < 0.001). Habitual drug experiences were 0.6%, 0.7%, and 2.0% in the normal, SCF, and SC groups, respectively (*p* < 0.001) (Table 3).

### 3.4. Mediating Effect of Stress Recognition on Smartphone Dependence and GAD

**Figure 1:**
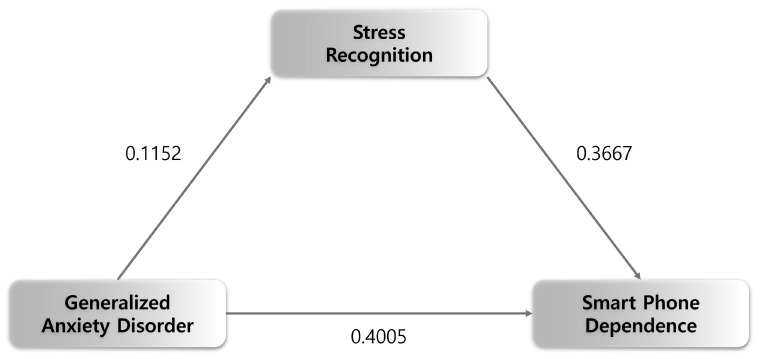
Moderated mediating effect of stress recognition and smartphone dependence and GAD.

Moving to the statistical analysis section, Figure 1 and Table 4 show that GAD had a significant effect on the recognition of stress (β = 0.115, *p* < 0.001), and recognition of stress had a significant effect on SPD (β = 0.367, *p* < 0.001). Thus, the relationship between GAD and SPD was mediated by perceived stress. Additionally, the total effect of SPD on GAD was β = 0.443 (*p* < 0.01); however, with the input of stress recognition as a variance, the direct effect of the SPD pathway on GAD was β = 0.401 (*p* < 0.01), confirming that the stress recognition was mediated. Table 4 shows that the indirect bootstrap analysis of GAD and SPD revealed that there is no 0 between LLCI and ULCI, confirming the indirect effect (Figure 1).

## 4. Discussion

This study analyzed the differences between normal, SCF, and SC according to the degree of SPD in 54,948 adolescents in middle and high schools and the mediating effect of GAD on SPD according to factors of stress recognition. This study analyzed efforts for weight control, health perception, body shape perception, happiness, and stress perception. It also examined the total effects and direct/indirect effects of subjective perception, subjective body shape, subjective happiness, subjective stress perception, and factors related to anxiety disorders, loneliness, depression, and suicidal thoughts on smartphone addiction. Stress recognition was confirmed to have a mediating effect on the effect of GAD on SPD. In a previous study, the relationship between GAD tendency and anxiety exhibited a similar pattern to that mediated by each maladaptive cognitive emotion control strategy [27]. The main result of the study was that both males and females tended to have higher grades and lower academic performance with smartphone overdependence and were perceived as unhealthy. In addition, their body type was more likely to be self-perceived as having “gained weight”, and they were more likely to be unhappy. As a result, GAD was associated with smartphone overdependence, anxiety, and increased negative parameters such as stress perception. It was found that the higher the dependence on a smartphone, the greater the body dissatisfaction, resulting in self-overestimating as “fat”.

It was found that the perception of stress during normal times increased proportionally with the level of smartphone dependence. High levels of loneliness, sadness, and despair were equally found. As the SPD increased, so did suicidal thoughts and substance abuse as a habit. It can be seen that the rate of loneliness in the previous year is proportional to dependence on smartphones. In the previous year, women were 54.6% more likely than men to have experienced sadness, hopelessness, and suicidal thoughts. Approximately 21.8% of men and 29.8% of women showed a high risk for sadness, hopelessness, and suicidal thoughts. However, as men’s smartphone dependence grew, the proportion of men who reported regular drug use (other than for treatment purposes) was found to be higher than that of women. It was confirmed that the usual stress perception was mediated finally by GAD and SPD.

The higher the degree of smartphone dependence, the lower the academic performance, which indicates that smartphones may be used more commonly for non-academic purposes [28]. Both men and women recognize that the higher their SPD, the worse their health and body shape, which was an indication of being overweight. As for happiness, the higher the SPD, the less happiness is acknowledged. It was hypothesized that a lack of interpersonal relationships and an increase in alone time were some of the factors contributing to the increased amount of smartphone usage [29]. The higher the adolescent level of internet use and SPA, the more problems were internalized, including depressive anxiety, somatic symptoms, and social withdrawal. Further, there were externalized problems, including carelessness, aggression, and delinquency [30]. Additionally, more problems with impulsivity and anger control were also observed [31]. Furthermore, the internet and SPA were found to cause serious problems throughout development, including hindering school adaptation, impeding social development [32,33], and increasing the risk of suicide [34].

Choi (Health and Welfare Forum) found that 35.4% of adolescents had stress, and women had a higher recognition of stress than men [35]. This recognition of stress was slightly higher than usual. It seems that the difference is a result of not only comparing simple stress but considering the level of SPD and stress simultaneously. Furthermore, as the grade level increases, the smartphone dependence and recognition of stress tend to increase with the same pattern [35]. The adolescence period is a time of psychological change, and adult roles change with physical development; thus, students experience many problems, including academic stress, anxiety, and problems with the opposite gender [36]. Therefore, the mental health problems experienced can affect students during the adolescence period and their overall adult life, creating a long-term impact; thus, it is necessary to urge social attention and make efforts to solve the problem.

Although policy and projects at the national level have a limited focus on adolescents, active intervention in mental health issues is required. In addition, it will be more effective if the efforts to identify blind spots in existing policies and to approach mental health issues in a holistic manner are combined by establishing a linkage system, and the roles between adolescent mental health policymakers are divided. Thus, if stress is typically reduced, GAD will be reduced, and the tendency to relieve stress with a smartphone will likewise be reduced. It will be effective if classes are organized at students’ levels, encouraging active class participation without excessive stress, and supporting various teaching–learning activities to reduce SPD in school. Furthermore, it is believed that adolescents should be helped by actively relieving their stress and anxiety. Another study examined the negative effects of smartphone use on male students as well as the contributing factors that lead to these adverse effects. The factors identified included academic performance, economic status, depression, suicide-related factors, happiness, subjective body shape, stress, and smartphone-usage time [37]. In this study, it was also found that smartphone addiction was influenced by various factors regardless of gender. These factors include academic performance, household income, happiness, health, weight control, anxiety, depression, and suicide. Adolescents’ engagement in regular exercise, maintaining a healthy diet, and following a consistent daily routine may help alleviate the negative effects of excessive smartphone use by addressing the underlying factors that contribute to anxiety and agitation. To alleviate anxiety and depressive emotions, there is an increasing reliance on smartphone due to the focus on communication through social networks and social networking sites (SNS) [38]. Furthermore, smartphone addiction strongly influences the depression of adolescents, and in severe cases, it can lead to suicidal thoughts [39,40]. Increased anxiety is closely related to stress, and research expects that reducing smartphone dependency and reducing exposure to the factors that mediate it will decrease these problematic behaviors.

The limitations of the study are as follows. This analysis is not representative of the entire adolescent population and should be interpreted with caution. There are limitations to subjective perception of stress and happiness variables as compared to objective scales. Analysis of cross-sectional public data has a limitation when attempting to establish a causal connection. Imminently, a longitudinal study will be conducted, and a subsequent follow-up study is required to verify the effect on health cognition and stress using an objective measure.

## 5. Conclusions

In conclusion, the habitual drug experience was higher in the SC group than in the normal and SCF groups. In the case of women, stress recognition was lower in the normal group than in the SCF and SCF groups. Experiences of loneliness, sadness, and hopelessness in the previous year were higher in the SC group than in the normal and SCF groups. Suicidal thoughts, suicidal plans, and suicidal attempts were more numerous in the SC group than in the normal and SCF groups. GAD had a significant effect on the recognition of stress, and the recognition of stress had a significant effect on SPD. Stress recognition was mediated in the relationship between generalized anxiety disorder and SPD. Furthermore, the total effect of SPD on GAD and the direct effect of the SPD pathway on GAD was significant; thus, stress recognition was mediated. Adolescents often experience large amounts of anxiety related to their academic performance and future employment. Excessive and inappropriate anxiety, however, can have negative effects on individuals’ health and well-being. It can lead to excessive stress and worry regarding their academic and employment concerns. Therefore, if stress is reduced, as suggested by this study, the psychological aspect of anxiety will also decrease. In other words, by preventing situations where anxiety and stress occur due to excessive smartphone use and by promoting the development of the character, knowledge, and interpersonal relationships that adolescents should possess, it is hoped that a foundation for helping adolescents becoming more proactive and positive individuals will be established.

## Figures and Tables

**Table 1:** Smartphone dependence and general characteristics.

Characteristics		Smartphone Dependence																	
		Males									Females								
		Normal ^a^		SCF ^b^		SC ^c^		Z ^+^/X^2 ++^	*p*	Post hoc **	Normal		SCF		SC		Z/X^2^	*p*	Post hoc
		N	%	N	%	N	%				N	%	N	%	N	%			
Height *		171.04	7.68	171.32	7.28	171.94	7.44	8.291	0.016	a < b,c	160.69	5.39	160.75	5.30	161.03	5.30	3.532	0.171	
Weight *		65.70	13.83	65.39	13.21	65.75	13.72	1.243	0.537		53.46	9.12	53.66	9.25	53.72	9.02	1.848	0.397	
Age *		15.07	1.76	15.26	1.73	15.54	1.74	84.024	0.000	a < b,c /b < c	15.01	1.77	15.21	1.69	15.26	1.66	83.191	0.000	a < b,c
Obesity	Normal	16728	76.1	3975	77.5	436	77.0	4.548	0.103		16,338	90.1	6034	89.9	903	91.2	1.738	0.419	
	obesity	5253	23.9	1155	22.5	130	23.0				1789	9.9	679	10.1	87	8.8			
Grade	High	3242	14.4	543	10.4	68	11.5	314.999	0.000		2213	11.9	582	8.4	88	8.5	472.409	0.000	
	High-middle	5465	24.3	1160	22.1	110	18.5				4940	26.5	1548	22.4	187	18.0			
	Middle	6681	29.7	1478	28.2	122	20.6				5998	32.2	2053	29.7	253	24.3			
	Middle-low	4859	21.6	1396	26.6	129	21.8				4111	22.0	1885	27.3	304	29.2			
	Low	2274	10.1	662	12.6	164	27.7				1390	7.5	835	12.1	208	20.0			
Class	Middle School 1	4275	19.0	753	14.4	70	11.8	131.916	0.000		3809	20.4	983	14.2	115	11.1	217.214	0.000	
	Middle School 2	3838	17.0	907	17.3	78	13.2				3368	18.1	1177	17.1	196	18.8			
	Middle School 3	3840	17.1	952	18.2	117	19.7				2966	15.9	1295	18.8	222	21.3			
	High School 1	3696	16.4	834	15.9	72	12.1				2965	15.9	1188	17.2	152	14.6			
	High School 2	3602	16.0	911	17.4	118	19.9				2829	15.2	1249	18.1	198	19.0			
	High School 3	3270	14.5	882	16.8	138	23.3				2715	14.6	1011	14.6	157	15.1			
Education (Father)	Middle School	243	2.0	56	1.9	12	4.0	11.137	0.025		249	2.1	76	1.7	22	3.1	17.380	0.002	
	High School	3493	28.6	794	26.7	90	29.7				3459	28.7	1395	31.1	188	26.7			
	University over	8462	69.4	2120	71.4	201	66.3				8351	69.3	3009	67.2	495	70.2			
Education (Mother)	Middle School	173	1.4	38	1.3	9	3.0	6.177	0.186		178	1.4	74	1.6	14	1.9	9.350	0.053	
	High School	3848	31.1	938	31.2	88	29.2				4337	34.1	1721	36.2	238	33.1			
	University over	8337	67.5	2034	67.6	204	67.8				8201	64.5	2963	62.3	468	65.0			
Family Income	High	2967	13.2	486	9.3	83	14.0	160.351	0.000		1935	10.4	480	7.0	88	8.5	141.675	0.000	
	High-middle	6476	28.8	1480	28.2	131	22.1				5098	27.3	1852	26.8	263	25.3			
	Middle	10272	45.6	2488	47.5	253	42.7				9378	50.3	3497	50.7	509	48.9			
	Middle-low	2261	10.0	654	12.5	80	13.5				1887	10.1	918	13.3	137	13.2			
	Low	545	2.4	131	2.5	46	7.8				354	1.9	156	2.3	43	4.1			

^a^, smartphone dependence was a normal group; ^b^, smartphone dependence was a self-control failure (SCF) group; ^c^, smartphone dependence was serious consequences (SC) group; * Mean ± standard deviation; ** post hoc, Bonferroni; N, frequency; %, percentage; ^+^ Kruskal–Wallis; ^++^ chi-square test (SCF, self-control failure; SC, serious consequences).

**Table 2:** Smartphone dependence and weight control; subjective recognition.

Characteristics		Smartphone Dependence																	
		Male									Female								
		Normal ^a^		SCF ^b^		SC ^c^		Z ^+^/X^2 ++^	*p*	Post hoc **	Normal		SCF		SC		Z ^+^/X^2 ++^	*p*	Post hoc
		N	%	N	%	N	%				N	%	N	%	N	%			
Subjective recognition *,^‡^	Health	4.08	0.88	3.81	0.91	3.64	1.12	476.732	0.000	a > b,c /b > c	3.84	0.87	3.59	0.88	3.45	1.02	499.798	0.000	a > b,c /b > c
	Body shape	3.13	1.01	3.09	1.08	3.11	1.21	4.992	0.082	a > b	3.24	0.88	3.28	0.94	3.28	1.01	17.324	0.000	a < b
	Happiness	3.99	0.94	3.67	0.95	3.37	1.17	624.812	0.000	a > b,c /b > c	3.80	0.95	3.50	0.93	3.28	1.06	674.696	0.000	a > b,c /b > c
Weight control effort * (the last month)	Not	10,642	47.3	2603	49.7	309	52.1	49.809	0.000		7712	41.3	3031	43.9	477	45.9	51.303	0.000	
	Loss	6339	28.1	1398	26.7	145	24.5				8002	42.9	2965	43.0	423	40.7			
	Gain	2756	12.2	712	13.6	91	15.3				457	2.5	166	2.4	38	3.7			
	Maintenance	2784	12.4	526	10.0	48	8.1				2481	13.3	741	10.7	102	9.8			

^a^, smartphone dependence was a normal group; ^b^, smartphone dependence was a self-control failure (SCF) group; ^c^, smartphone dependence was serious consequences (SC) group; ^+^ Chi-square test; ^++^ Chi-square test for nominal variable; ** ANOVA Post hoc test was Bonferroni; SCF, self-control failure; SC, serious consequences; ^‡^ average and standard deviation; * subjective recognition; Health: “Do you think you are healthy?”; Body shape: satisfaction with one’s body shape; Happiness: “Do you think you are happy?”. Subjective recognition, health, and happiness were examined using a Likert scale with 5 points on a scale from 1 (very not) to 5 (very good) and body shape on a scale from 1 (very thin) to 5 (very fat), where there was effort to control weight in the last 30 days.

**Table 3:** Smartphone dependence and mental health.

Characteristics		Smartphone Dependence																	
		Male									Female								
		Normal ^a^		SCF ^b^		SC ^c^		X^2^	*p*	Post hoc **	Normal		SCF		SC		X^2^	*p*	Post hoc **
		N	%	N	%	N	%				N	%	N	%	N	%			
Stress recognition		2.95	0.96	3.23	0.85	3.54	1.04	556.246	0.000	c > a,b /b > a	3.24	0.90	3.52	0.86	3.85	0.93	823.501	0.000	c > a,b /b > a
GAD		9.67	3.63	11.65	4.40	14.10	6.23	1586.014	0.000	c > a,b /b > a	10.98	4.21	13.14	4.74	16.02	6.04	2011.772	0.000	c > a,b /b > a
Loneliness experience *^/^**^/^***		2.13	1.03	2.54	1.02	2.83	1.26	843.549	0.000	a < b,c /b < c	2.47	1.04	2.90	0.98	3.18	1.13	1158.431	0.000	a < b,c /b < c
Sadness and despair **^/^***	No	18,507	82.2	3861	73.7	352	59.4	356.125	0.000		13,740	73.7	4176	60.5	472	45.4	695.836	0.000	
	Yes	4014	17.8	1378	26.3	241	40.6				4912	26.3	2727	39.5	568	54.6			
Suicidal thoughts **^/^***	No	20,983	93.2	4652	88.8	464	78.2	268.896	0.000		16,510	88.5	5630	81.6	730	70.2	426.836	0.000	
	Yes	1538	6.8	587	11.2	129	21.8				2142	11.5	1273	18.4	310	29.8			
Suicidal plans **^/^***	No	21,974	97.6	5068	96.7	541	91.2	95.219	0.000		17,954	96.3	6524	94.5	934	89.8	120.245	0.000	
	Yes	547	2.4	171	3.3	52	8.8				698	3.7	379	5.5	106	10.2			
Suicidal attempts **^/^***	No	22,240	98.8	5167	98.6	564	95.1	57.716	0.000		18,212	97.6	6670	96.6	974	93.7	70.252	0.000	
	Yes	281	1.2	72	1.4	29	4.9				440	2.4	233	3.4	66	6.3			
Habitual drug experience	No	22,360	99.3	5190	99.1	576	97.1	70.137	0.000		18,545	99.4	6853	99.3	1019	98.0	77.945	0.000	
	Yes	161	0.7	49	0.9	17	2.9				107	0.6	50	0.7	21	2.0			

^a^, smartphone dependence was a normal group; ^b^, smartphone dependence was a self-control failure (SCF) group; ^c^, smartphone dependence was serious consequences (SC) group; * Reverse coding; ** 1 year ago; *** except for therapeutic; SCF, self-control failure; SC, serious consequences.

**Table 4:** Mediating effect of stress recognition on smartphone dependence and GAD.

Characteristics		*β*		se		t	*p*	LLCI	ULCI
Mediate variable model (Dependent variable: SR)									
Constant		1.9135		0.0091		209.2416	0.0000	1.8956	1.9314
GAD		0.1152		0.0008		148.0074	0.0000	0.1137	0.1167
Dependent variable model (Dependent variable: SPD)									
Constant		12.9950		0.0915		142.0354	0.0000	12.8157	13.1744
GAD		0.4005		0.0069		58.2905	0.0000	0.3870	0.4140
MSTR		0.3667		0.0318		11.5178	0.0000	0.3043	0.4291
**Effect**			***β***			**se**	**LLCI**		**ULCI**
Total effect			0.4427			0.0058	0.4313		0.4541
Direct effect			0.4005			0.0069	0.3870		0.4140
Indirect effect			0.0422			0.0039	0.0346		0.0501

## Data Availability

No new data were created or analyzed in this study. Data sharing is not applicable to this article.
